# Supplementary material for: Streptolysin O accelerates the conversion of plasminogen to plasmin
Source: Nat Commun. 2024 Nov 25;15:10212. doi: 10.1038/s41467-024-54173-6 (PMC11589678; doi:10.1038/s41467-024-54173-6)
Supplement: Supplementary file 1 — Supplementary Information [file 41467_2024_54173_MOESM1_ESM.pdf]

Supplementary Data Figure 1: Tang et al

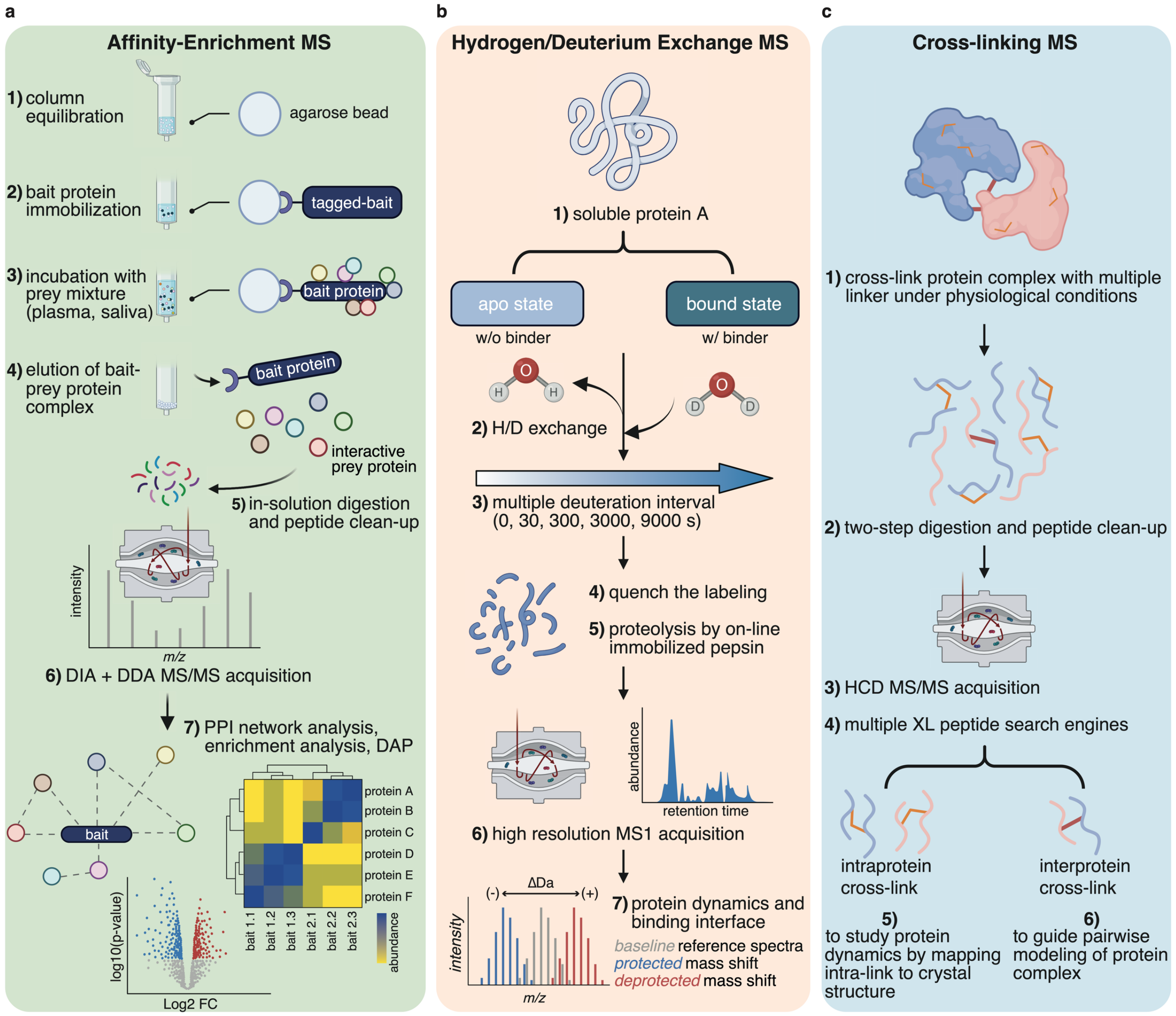

Supplementary Figure 1 I Integrative MS-based proteomics workflow for studying host-pathogen interactions
The integrative MS-based proteomics strategy applied in this study includes **a)** Affinity-Enrichment, **b)** Hydrogen/Deuterium Exchange, and **c)** Cross-linking Mass Spectrometry workflows. Under near-physiological conditions, AE-MS identifies prey proteins from mixtures that interact with the bacterial bait proteins. Two orthogonal structural proteomics methods, HDX-MS and XL-MS, were used to characterize the indicated bait-prey protein-protein interactions in terms of binding interfaces and to investigate protein dynamics in solution. DAP: differentially abundant protein. Panel **a-c** created with Biorender.com.

Supplementary Data Figure 2: Tang et al

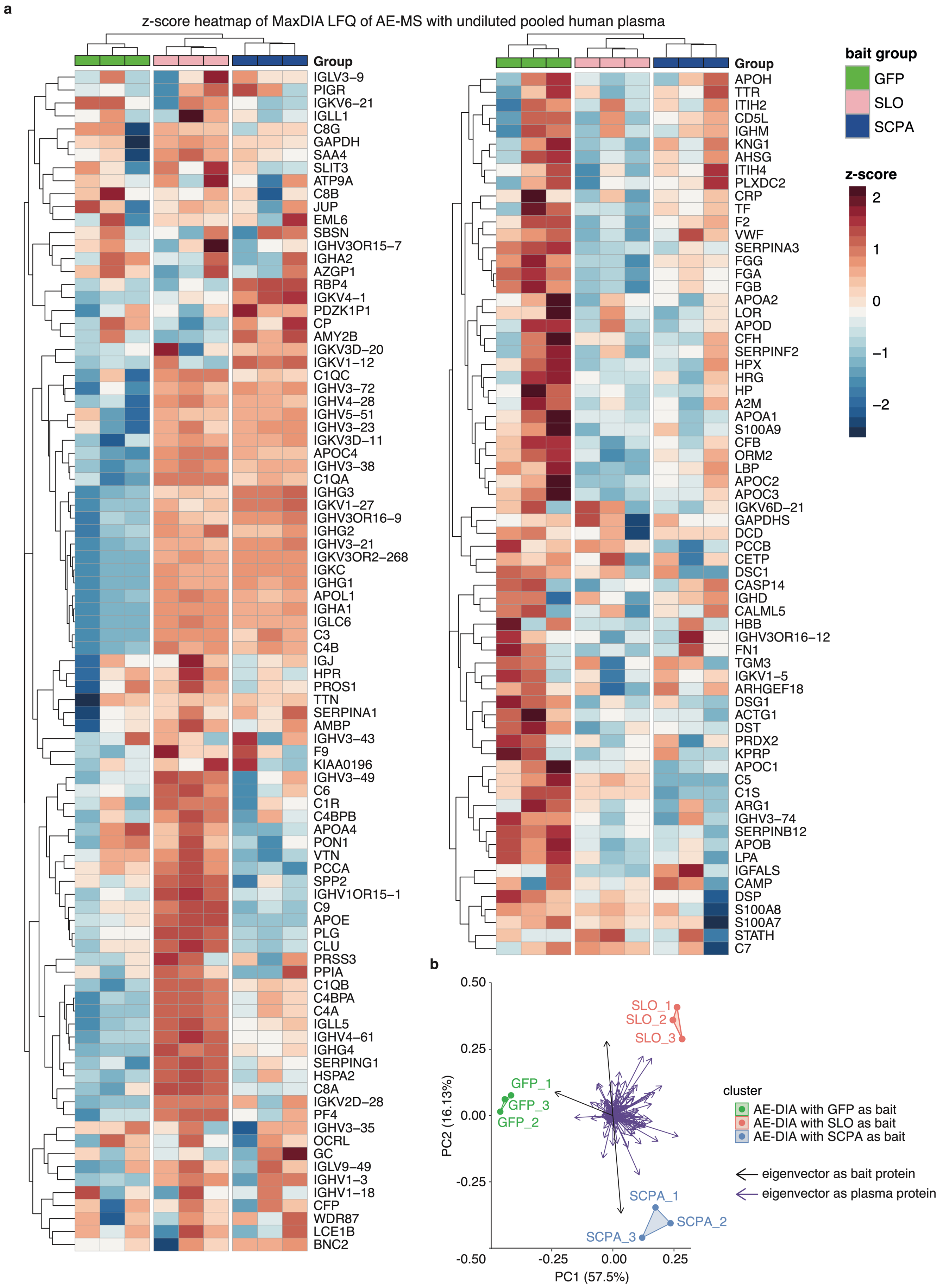

Supplementary Figure 2 | Comprehensive proteomics profiling of identified human plasma proteins through AE-MS DIA

a) Clustered heatmap of intensity z-scores, calculated by row-level normalization of the log_2-transformed LFQ intensity of identified prey protein across experimental replicates in different bait groups. The colour scale indicates relative enrichment (red) or reduction (blue) of the prey protein group. **b)** Principal component analysis (PCA) plot clusters nine AE-MS pull-down samples based on protein abundances, with the contribution from corresponding bait protein marked by black arrows. Source data are provided as a Source Data file.

Supplementary Data Figure 3: Tang et al

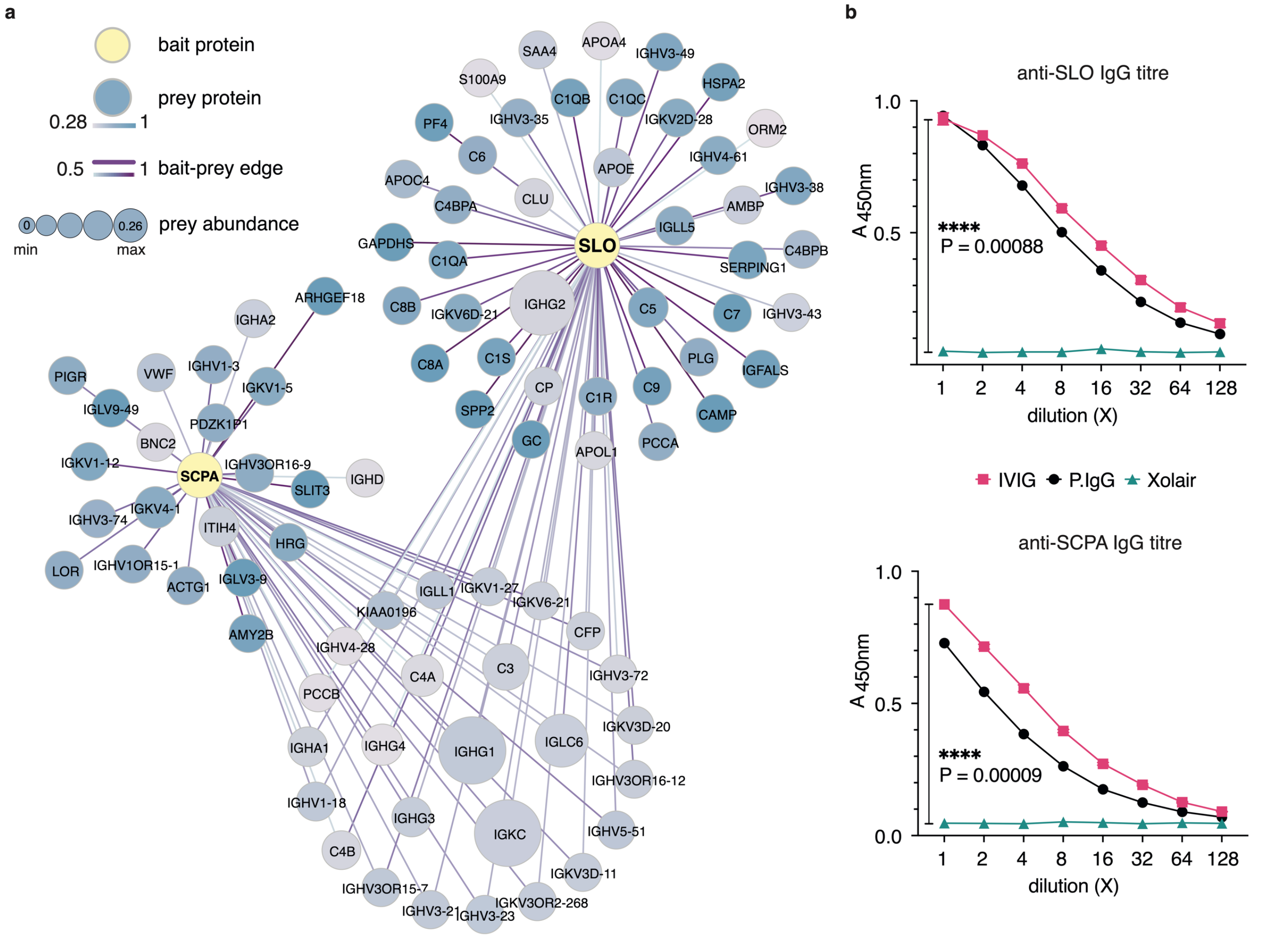

Supplementary Figure 3 | Combined PPI networks of SLO/SCPA and plasma proteins, alongside circulating specific IgGs in human plasma

a) This combined network summarises the interactions between tagged SLO/tagged SCPA and human plasma proteins. Nodes represent proteins, with the node size and colour intensity reflecting the abundance and specificity of the isolated prey proteins, respectively. Connecting edges, indicative of protein-protein interactions (PPI), are coloured according to calculated MiST scores (> 0.5). **b)** ELISA measurements of IgG titres against SLO (streptolysin O) and SCPA (C5a peptidase) across different IgG samples. Absorbance values at 450 nm (A_{450nm}) are shown for IVIG (pooled healthy human IgG isolates), P.IgG (IgGs from a patient recovering from a recent GAS infection), and Xolair (negative control, a monoclonal IgG1 specific to IgE). Source data are provided as a Source Data file.

Supplementary Data Figure 4: Tang et al

a

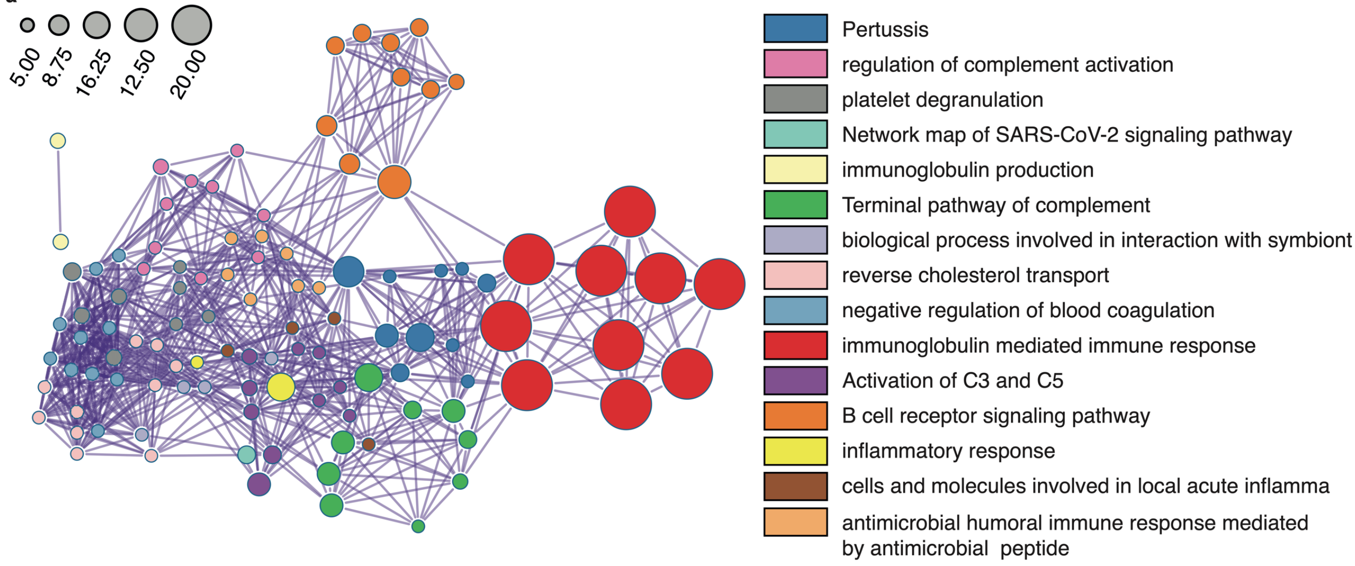

b

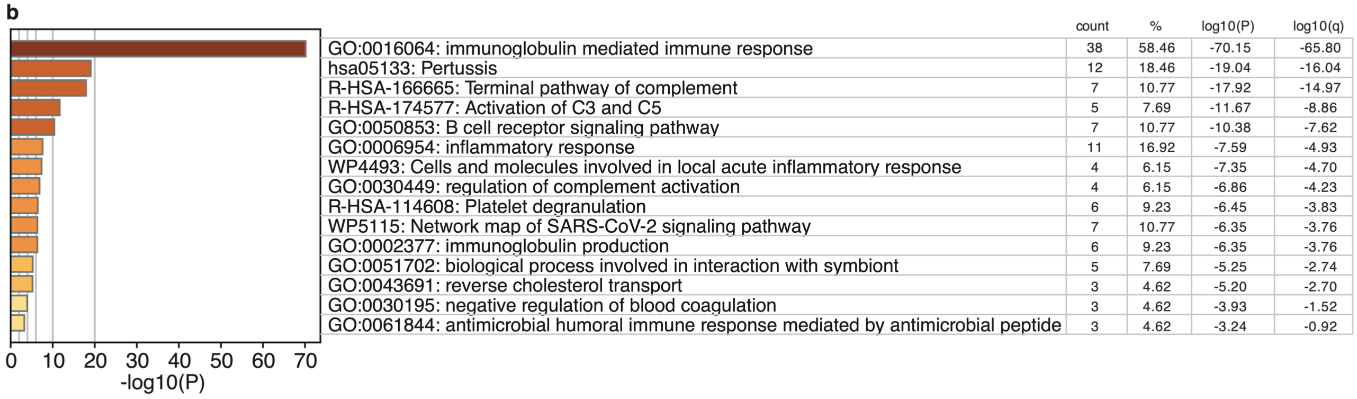

	count	%	log10(P)	log10(q)
GO:0016064: immunoglobulin mediated immune response	38	58.46	-70.15	-65.80
hsa05133: Pertussis	12	18.46	-19.04	-16.04
R-HSA-166665: Terminal pathway of complement	7	10.77	-17.92	-14.97
R-HSA-174577: Activation of C3 and C5	5	7.69	-11.67	-8.86
GO:0050853: B cell receptor signaling pathway	7	10.77	-10.38	-7.62
GO:0006954: inflammatory response	11	16.92	-7.59	-4.93
WP4493: Cells and molecules involved in local acute inflammatory response	4	6.15	-7.35	-4.70
GO:0030449: regulation of complement activation	4	6.15	-6.86	-4.23
R-HSA-114608: Platelet degranulation	6	9.23	-6.45	-3.83
WP5115: Network map of SARS-CoV-2 signaling pathway	7	10.77	-6.35	-3.76
GO:0002377: immunoglobulin production	6	9.23	-6.35	-3.76
GO:0051702: biological process involved in interaction with symbiont	5	7.69	-5.25	-2.74
GO:0043691: reverse cholesterol transport	3	4.62	-5.20	-2.70
GO:0030195: negative regulation of blood coagulation	3	4.62	-3.93	-1.52
GO:0061844: antimicrobial humoral immune response mediated by antimicrobial peptide	3	4.62	-3.24	-0.92

Supplementary Figure 4 l Over-representation analysis of SLO-enriched proteins

68 putative SLO-interactive plasma proteins (based on MiST score > 0.5) were subjected to over-representation analysis. **a)** The clustered network visualizes the complete list of enriched terms from the GO:BP (Gene Ontology Biological Process), KEGG pathway, Reactome, and WikiPathways databases. The most representative term from the top 15 enriched clusters is displayed. **b)** The bar graph illustrates these 15 representative enriched terms, with colours indicating the P-values, coupled with the count as the number of genes from the input list associated with the given term, the % representing the proportion of input genes in each term, and both the $\log_{10}$-transformed P-values log10(P) and multi-test adjusted P-values $\log_{10}(q)$ for statistical context. Source data are provided as a Source Data file.

Supplementary Data Figure 5: Tang et al

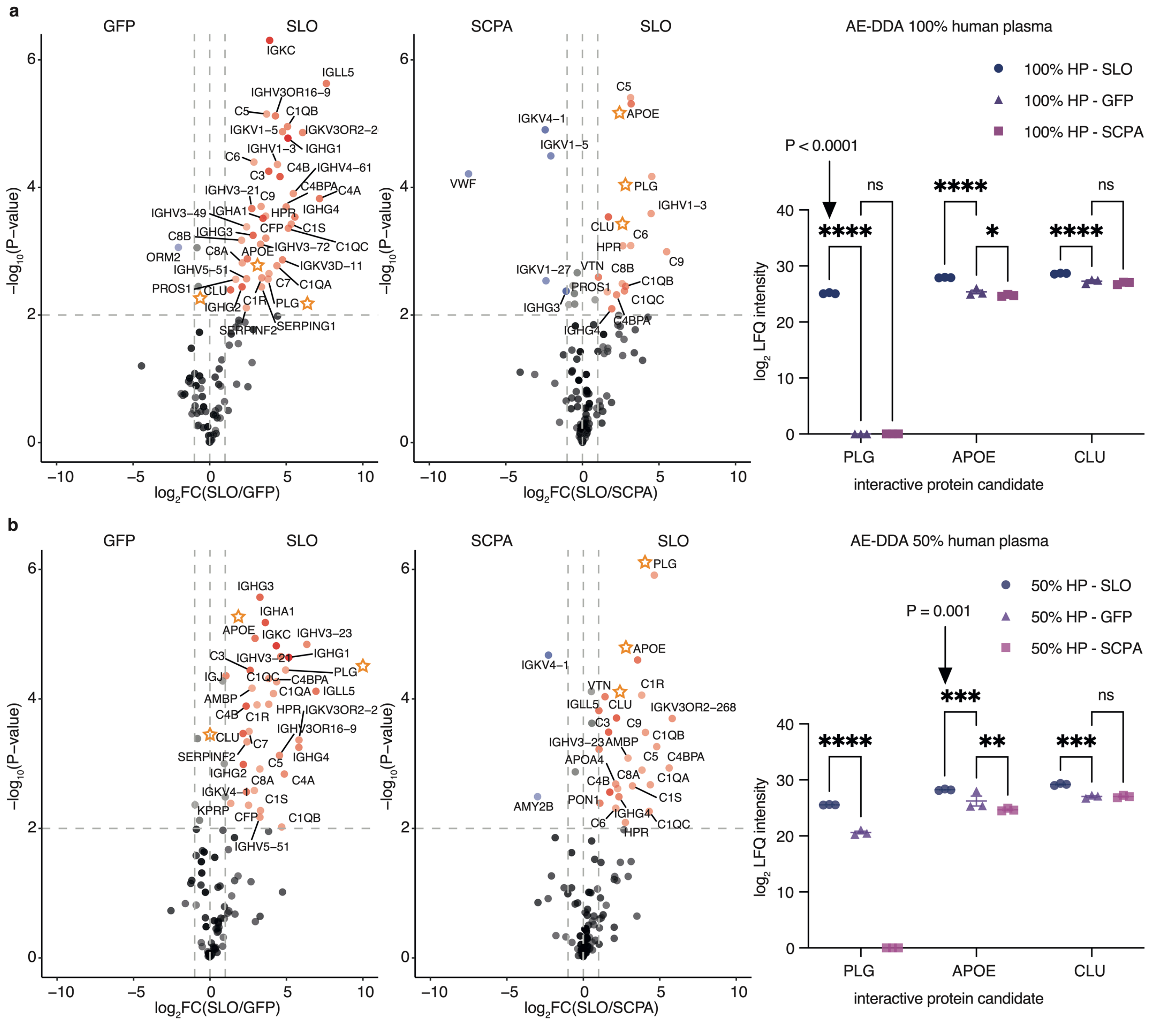

Supplementary Figure 5 | AE-DDA MS differential analysis using diluted human plasma as prey mixture Pooled HP (human plasma) used in either undiluted (100% HP) or diluted to 50% with PBS, was subjected to AE-MS in three independent experiments. Proteins eluted were analysed by DDA (data dependent acquisition) MS and searched against the human reference proteome database. Prey protein abundances in SLO bait group were compared against bait GFP or SCPA group. Fold change of normalized LFQ data and FDR-controlled multiple t-tests identified proteins differentially enriched by SLO, coloured in red. Volcano plots were plotted for **a**, left**)** undiluted (100% HP) and **b**, left**)** 50% diluted HP group. Two-way ANOVA (Tukey's post-hoc test) on log2-transformed LFQ intensities highlighted the top three putative SLO-interactive plasma proteins: plasminogen (PLG), apolipoprotein E (APOE), and clusterin (CLU). **a-b**, right**)** Individual dot plots display the mean and standard deviation of candidate protein intensities, with the colour coding indicating the dilution factor. Source data are provided as a Source Data file.

Supplementary Data Figure 6: Tang et al

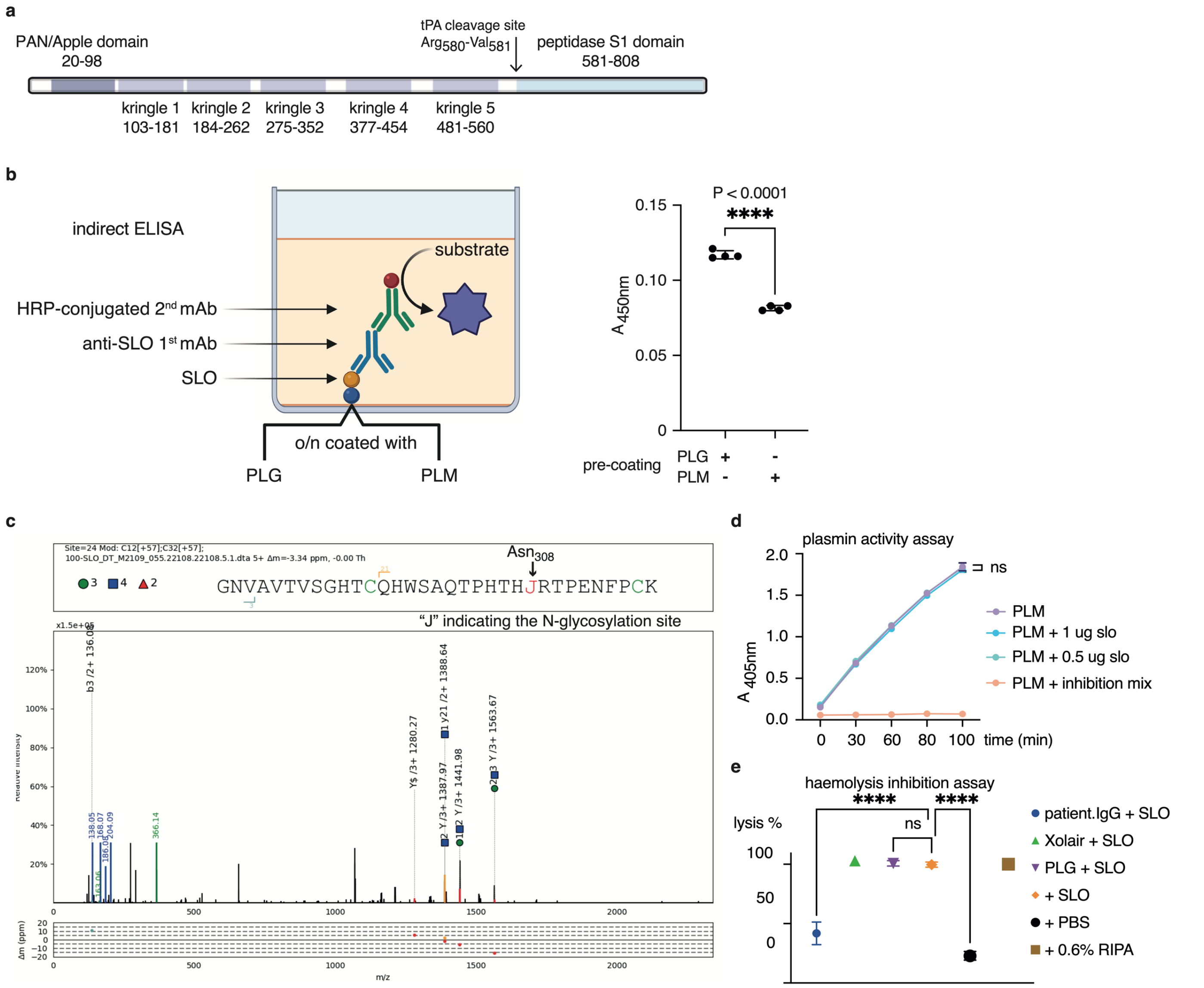

Supplementary Figure 6 I Domains of PLG, ELISA, targeted glycoproteomics, PLM activity assay, and SLO-mediated haemolysis assay

a) The schematic presentation of the full length PLG protein (UniProtID: P00747), with seven consecutive domains with residue numbers and tPA cleavage site annotated. **b)** An indirect ELISA was used to quantify binding of SLO to PLG/PLM (plasmin), with higher absorbance values indicating relatively stronger binding. Background signal was subtracted. **c)** MS/MS spectra of a representative PLG glycopeptide identified in SLO-enriched plasma samples. The analysis, performed with pGlyco 3.0 and visualized via pGlycoLabel, revealed glycosylation at Asn_{308} on PLG, indicative of type I PLG. **d)** Plasmin activity assay was performed where SLO and plasmin (PLM) were incubated for 30 minutes before adding a chromogenic plasmin substrate. The graph plots absorbance at 405 nm against the time of development, including three replicates per condition. **e)** SLO-mediated haemolysis inhibition assay was conducted where SLO, pre-incubated with different types of samples, was activated by TCEP and then added to the diluted suspension of sheep red blood cells. RIPA, a cell lysis detergent, acted as the positive control. Normalized haemolysis percentage was determined based on haemoglobin levels of collected supernatant after centrifuge in the experimental groups. Statistical differences among groups were evaluated using one-way ANOVA, with significance levels marked as ns (not significant), *,**, ***, and **** for P-values < 0.1, < 0.01, < 0.001, and < 0.0001, respectively. **b** created with Biorender.com. Source data are provided as a Source Data file.

Supplementary Data Figure 7: Tang et al

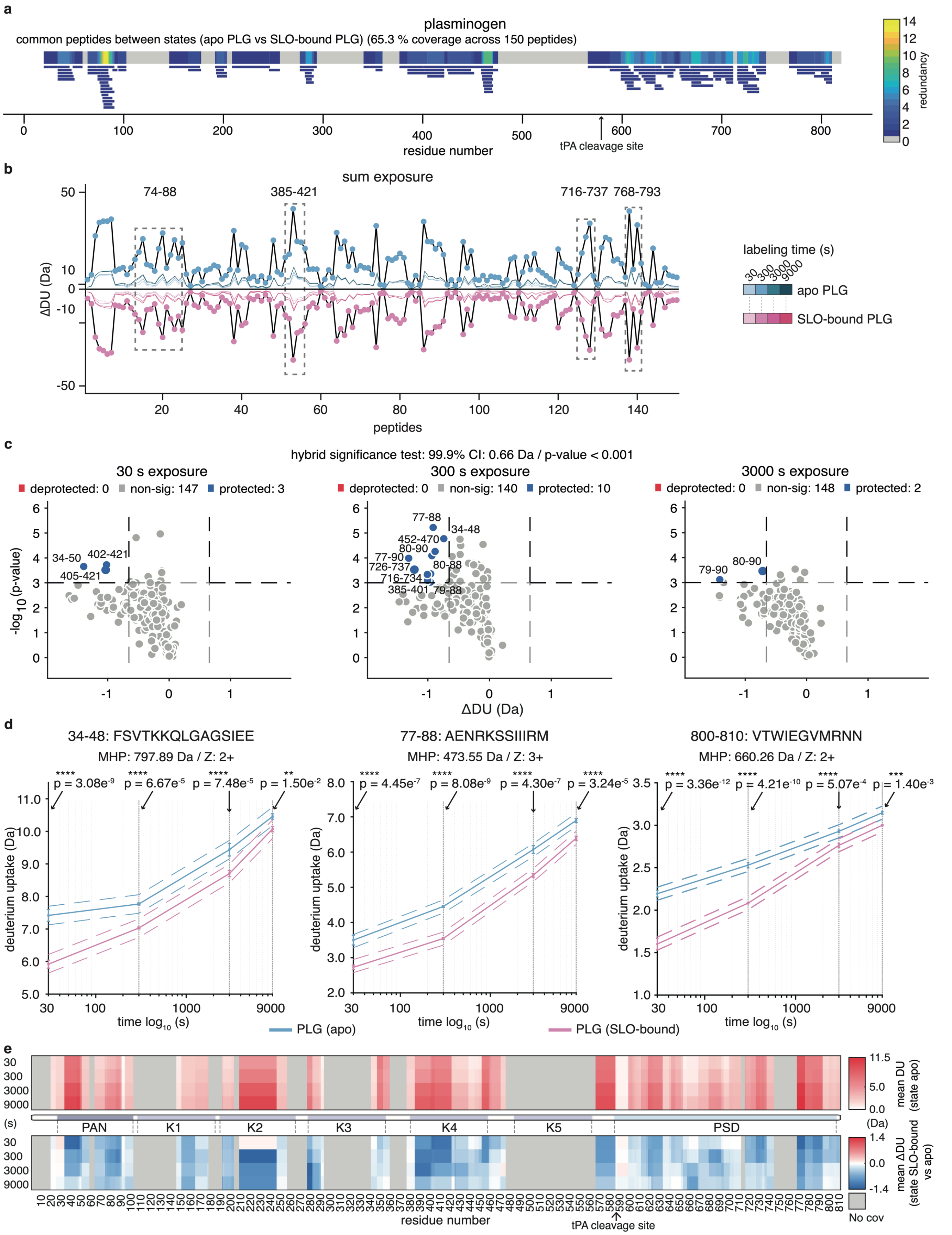

Supplementary Figure 7 | Protected regions and protein dynamics of SLO-bound PLG revealed by HDX-MS

a) A redundancy map of 150 common peptides identified in both states of apo PLG and the SLO-bound PLG in HDX-MS, with bars below representing peptides and the colour gradient indicating residue redundancy. **b)** The butterfly plot

aggregates the summed deuterium uptake of all identified PLG peptides over the four labelling intervals of two states (apo and SLO-bound), with every common peptide shown as a dot. A dashed-line box highlights protected regions of PLG upon SLO binding, annotated with residue numbers. Overall, the ΔDU between apo PLG and SLO-bound PLG were marginal, with notable exceptions in the peptide regions spanning residues 74-88 in PAN, 385-421 in K4 domain, and 716-737 and 768-793 in PSD. **c)** Volcano plots display peptides with significant changes in deuterium uptake at 30, 300, and 3000 s where peptides are shown as dots and coloured based on the level of protection/deprotection. **d)** Kinetic plots for three protected peptides in PAN and PSD region, with significance testing across four labelling times, color-coded to different states. Significance levels are marked, with P-values annotated. MHP refers to theoretical molecular weight of the peptide, Z to charge state. **e)** The top panel barcode plot displays the mean deuterium uptake of PLG residues in apo state while the bottom panel represent mean differential deuterium uptake in SLO-bound state compared to the apo, with the colour gradient indicating the change level, as a function of labelling time. Source data are provided as a Source Data file.

Supplementary Data Figure 8: Tang et al

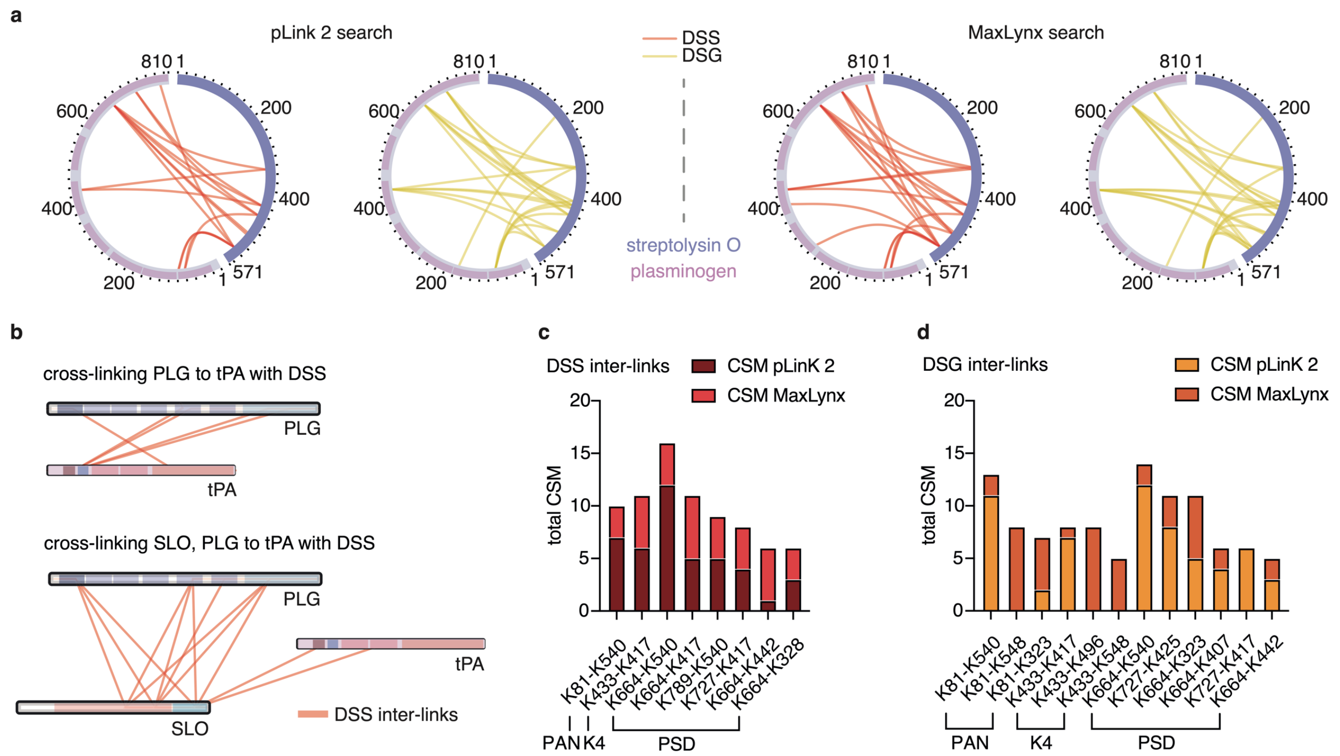

Supplementary Figure 8 I Comparative analysis using two XL-MS search engines, linkage maps of inter-links for PLG-tPA and PLG-SLO-tPA, alongside CSM count summary of unique inter-links

a) Circular maps present unique interprotein cross-links between plasminogen and streptolysin O, identified by either pLink 2 or MaxLynx in both DSS and DSG cross-linked datasets. **b)** Two linkage maps depict the DSS interprotein cross-links, displaying the interactions within the PLG-tPA and PLG-SLO-tPA complexes. **c-d)** Summary of CSM count for identified interprotein cross-links found between PLG and SLO. PLG domains containing cross-linked sites were annotated along x-axis. Source data are provided as a Source Data file.

Supplementary Data Figure 9: Tang et al

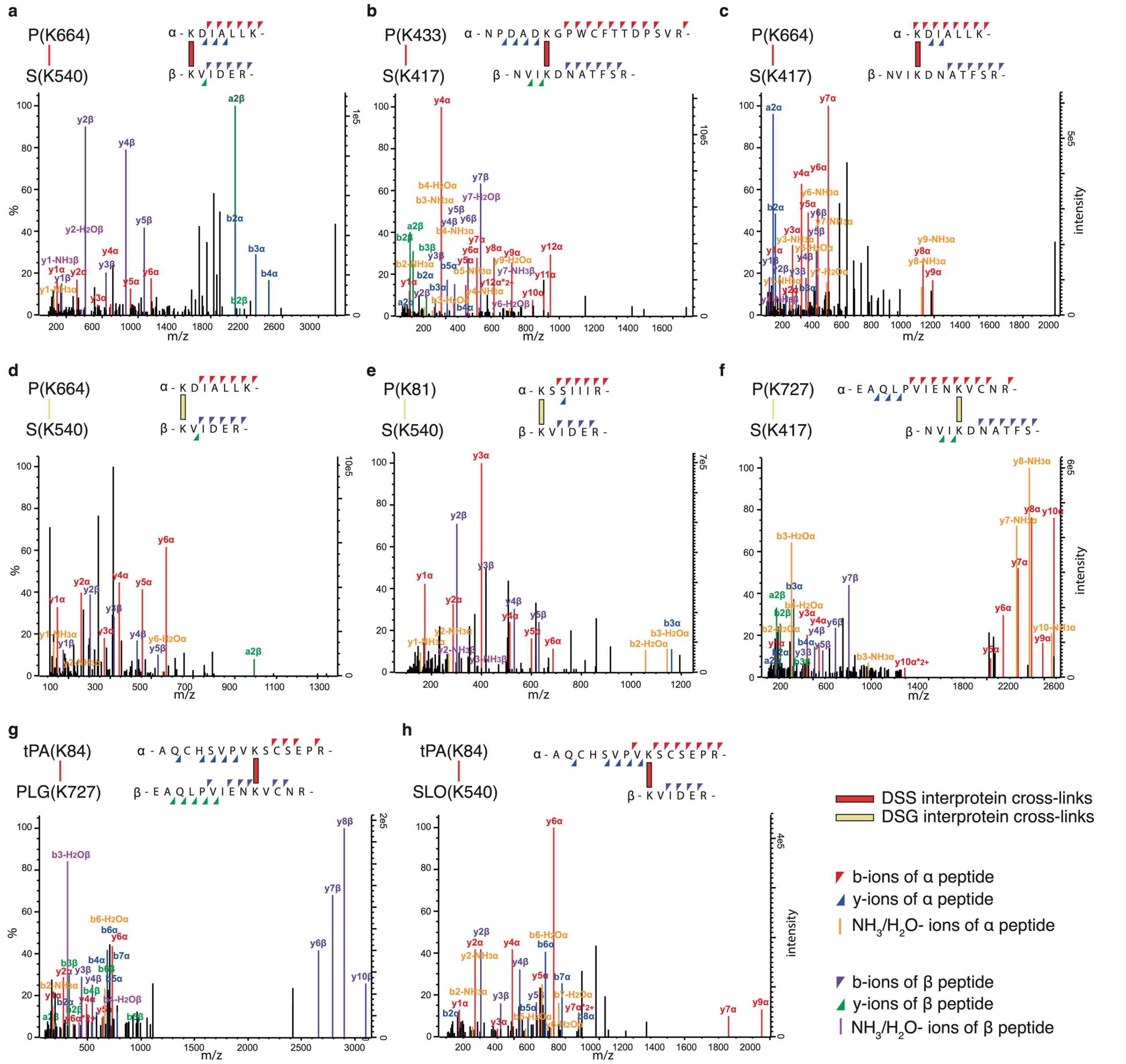

Supplementary Figure 9 I Annotated MS/MS spectra of cross-linked peptide pairs in PLG-SLO, PLG-tPA, and PLG-SLO-tPA interactions

Representative MS/MS spectra for **a-c)** DSS or **d-f)** DSG cross-linked peptide pairs between PLG domains (PSD, Kringle 4, PAN) and SLO domains (D4 and D3). **g)** Representative MS/MS spectra of cross-linked peptide pairs between tPA (EGF-like domain) and PLG PSD; and **h)** tPA (EGF-like domain) and SLO domain 4. All matched fragmented ions are annotated and coloured according to the legend.

Supplementary Data Figure 10: Tang et al

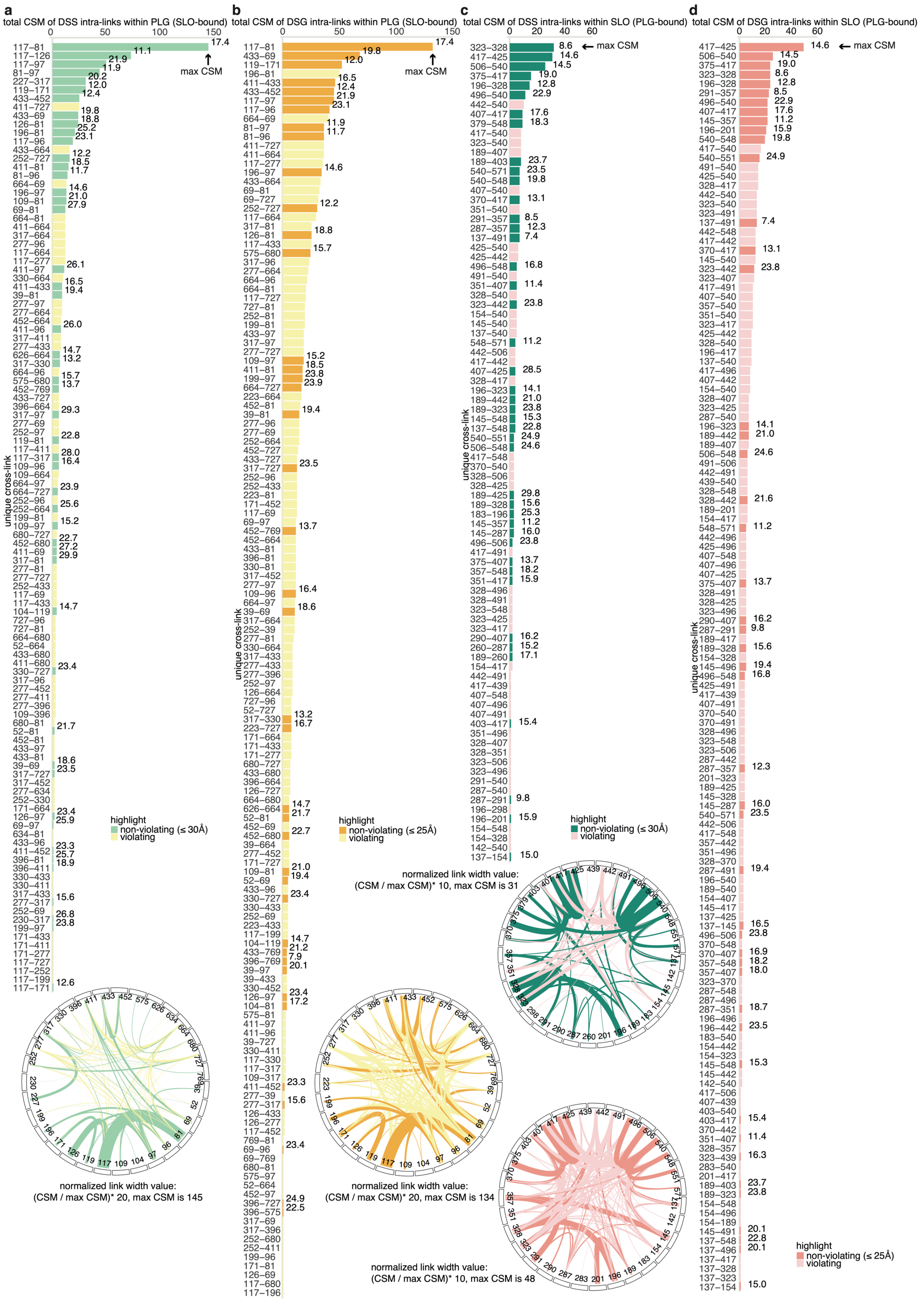

Supplementary Figure 10 lCSM summary of intra-links found in PLG or SLO during the interaction

a-d) Bar plots present CSM (cross-linked spectrum match) count summarized at the unique cross-linked site level. Accompanying circos plots illustrate the distribution of these cross-linked sites along protein sequences, with edge widths proportionally indicating respective CSM counts. Cα-Cα distance was measured by mapping the corresponding non-overlapping-associated intra-link onto the reference structure and annotated accordingly. Source data are provided as a Source Data file.

Supplementary Data Figure 11: Tang et al

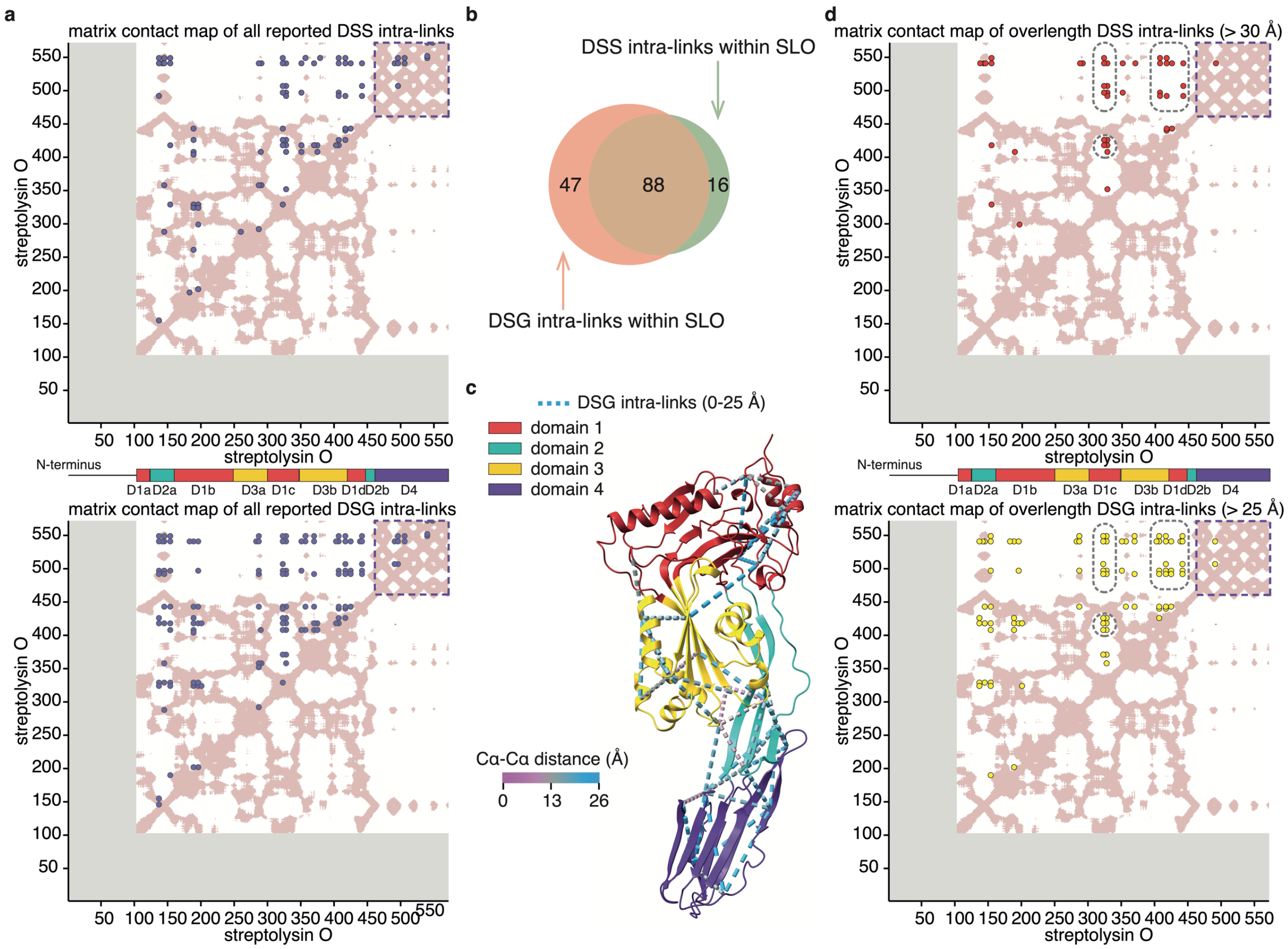

Supplementary Figure 11 | Matrix contact map of intra-links found within SLO against its reference static structure

a) Two matrix map presents all identified intra-links as dots based on the reference SLO crystal structure (PDB: 4HSC). White background represents resolved residues in the input structure, grey for absence and pink for cross-links with a Cα-Cα distance ≤ 25 Å. The structured domains of SLO (103-571), are colour coded according to the legend. **b)** A Venn diagram of the overlapping and unique intra-linked sites found within SLO from both the DSS and DSG cross-linked datasets. **c)** A crystal structure of SLO is coloured by domain, with consistent DSG intra-links (Cα-Cα distance ≤ 25 Å) displayed in dot-line style pseudo bond. **d)** Two matrix maps showing the over-length intra-links with a Cα-Cα distance > 30 Å for DSS and Cα-Cα distance > 25 Å for DSG as dots against the SLO structure. Source data are provided as a Source Data file.

Supplementary Data Figure 12: Tang et al

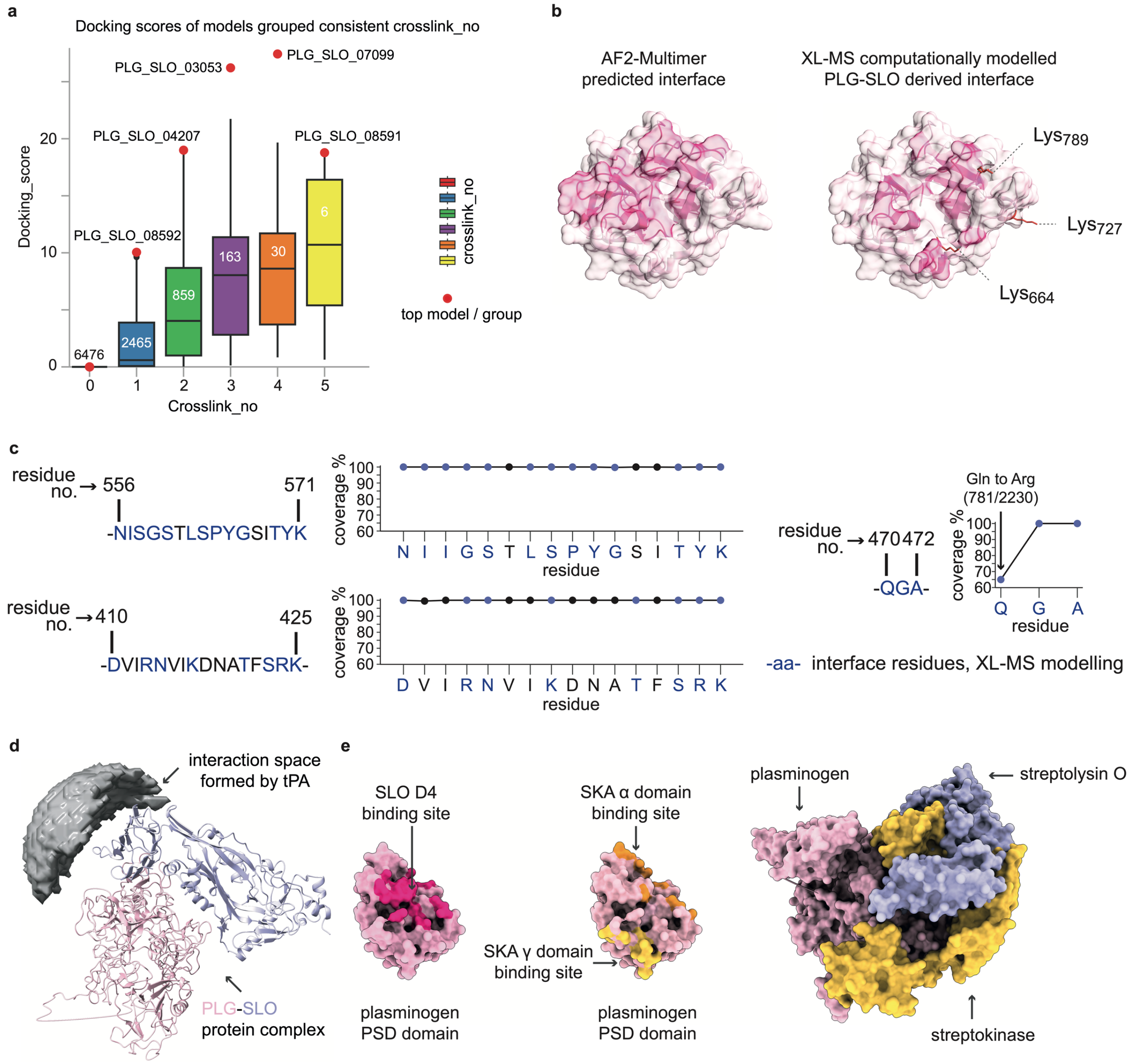

Supplementary Figure 12 l Summary of computational modelling analysis, conservation of PLG-binding motifs on SLO, and the side-by-side comparison of interaction sites on PLG

a) The box plot shows the distribution of docking scores across all pairwise PLG-SLO models using the bent SLO conformer, grouped by the number of consistent inter-links. The numbers of models that accommodate the cross-links are annotated across the groups. **b)** A comparison of SLO-binding sites on PLG PSD shown in surface representation between models predicted by AlphaFold2-Multimer and XL-MS computationally modelling. **c)** Three PLG-binding interfaces on SLO protein were identified in the top-ranked predicted model and analysed for sequence variability among 2230 high-quality GAS genomes downloaded from The Bacterial and Viral Bioinformatics Resource Centre. Streptolysin O sequence (Uniprot ID: P0DF96) is set as the reference. Interface residues derived from the modelled complex are coloured in blue and highlighted in bold. **d)** The interaction space between tPA and the PLG-SLO complex for all tPA-PLG-SLO conformations consistent with the two reported cross-link distance constraints between tPA and SLO. **e)** Left: the side-by-side comparison illustrates the SLO-binding interface on the PLG peptidase S1 domain, alongside the reported binding sites for the SKA α and γ domains. On the right, the co-crystal structure of PSD-SKA complex is superimposed onto the modelled PLG-SLO complex. Source data are provided as a Source Data file.

bait-prey association	MiST		bait-prey association	MiST
SLO (interacts with) IGHV3-49	0.85465		SCPA (interacts with) IGLV9-49	0.98713
SLO (interacts with) IGHV3-38	0.83959		SCPA (interacts with) IGLV3-9	0.88015
SLO (interacts with) IGKV6D-21	0.80797		SCPA (interacts with) IGKV1-12	0.84374
SLO (interacts with) IGKV2D-28	0.80559		SCPA (interacts with) IGKV1-5	0.80931
SLO (interacts with) IGHV4-61	0.7931		SCPA (interacts with) IGKV4-1	0.79063
SLO (interacts with) IGLL5	0.77996		SCPA (interacts with) IGHV3OR16-9	0.7852
			SCPA (interacts with) IGHV1OR15-1	0.76964
			SCPA (interacts with) IGHV1-3	0.7573

Supplementary Table 1 I Specifically enriched IgG chains by SLO or SCPA bait
The table lists all identified IgG chains specifically enriched in SLO or SCPA bait groups. MiST cut-off was raised to 0.75 instead to reduce false positive rate.

P-S; DSS XL-MS	CSM_pLink 2	rank_ pL	CSM_ MaxLynx	rank_ Ma	SUM CSM	SUM rank		P-S; DSG XL-MS	CSM_pLink 2	rank_ pL	CSM_ MaxLynx	rank_ Ma	SUM CSM	SUM rank
K664-K540	12	1	4	3	16	4		K664-K540	12	1	2	5	14	6
K664-K417	5	4	6	1	11	5		K81-K540	11	2	2	5	13	7
K433-K417	6	3	5	2	11	5		K727-K425	8	3	3	4	11	7
K81-K540	7	2	3	4	10	6		K664-K323	5	6	6	2	11	8
K789-K540	5	4	4	3	9	7		K433-K417	7	4	1	6	8	10
K727-K417	4	5	4	3	8	8		K81-K548		11	8	1	8	12
K664-K442	1	8	5	2	6	10		K433-K496		11	8	1	8	12
K664-K328	3	6	3	4	6	10		K81-K323	2	9	5	3	7	12
K664-K491	2	7	2	5	4	12		K664-K407	4	7	2	5	6	12
K81-K496		9	3	4	3	13		K727-K417	6	5		7	6	12
K277-K540		9	3	4	3	13		K664-K442	3	8	2	5	5	13
K396-K540		9	3	4	3	13		K433-K548		11	5	3	5	14
K433-K328	2	7	1	6	3	13		K81-K417	1	10	3	4	4	14
K433-K323		9	2	5	2	14		K81-K491	1	10	3	4	4	14
K727-K323		9	2	5	2	14		K433-K425	1	10	3	4	4	14
K727-K540		9	2	5	2	14		K727-K328	1	10	3	4	4	14
K789-K496		9	2	5	2	14		K81-K442	2	9	2	5	4	14
K727-K442	1	8	1	6	2	14		K81-K407	3	8	1	6	4	14
K664-K496	1	8	1	6	2	14		K171-K189	1	10	2	5	3	15
K97-K540	1	8	1	6	2	14		K433-K540	1	10	2	5	3	15
K96-K540	1	8	1	6	2	14		K433-K491	2	9	1	6	3	15
K81-K442	1	8	1	6	2	14		K727-K442	2	9	1	6	3	15
								K433-K323	3	8		7	3	15
								K664-K548		11	2	5	2	16
								K664-K417	1	10	1	6	2	16

Supplementary Table 2 I CSM Summary of inter-protein cross-links between PLG and SLO
The table lists all identified DSS and DSG inter-protein cross-linked sites (with at least 2 CSM evidence) between P (plasminogen) and S (streptolysin O), ranked separately by the total number of CSM reported by pLink 2 (rank_pL) and MaxLynx (rank_Ma) search engines, with a CSM sum (SUM CSM) and an aggregated ranking order (SUM rank).

Cross-link number	peptide1	peptide2	protein1	protein2	Nζ-Nζ distance (Å)	MS/MS Spectrum no. (linker) in Sup. Fig.9
**1)**, P_K664-S_K540	**K**DIALLK	**K**VIDER	plasminogen	streptolysin O	37.2 (top1); 23.5 (2nd); 33.9 (AF2M)	**a (DSS)**
**2)**, P_K664-S_K417	**K**DIALLK	NVI**K**DNATFSR	plasminogen	streptolysin O	18.0 (top1)	**c (DSS)**
**3)**, P_K789-S_K540	PN**K**PGVYVR	**K**VIDER	plasminogen	streptolysin O	29.5 (top1)	
**4)**, P_K727-S_K417	EAQLPVIEN**K**VCNR	NVI**K**DNATFSR	plasminogen	streptolysin O	13.4 (top1)	**f (DSG)**
**5)**, P_K575-S_K540	PQVEP**K**K	**K**VIDER	plasminogen	streptolysin O	34.8 (top1); 37.8 (2nd)	
**6)**, P_K433-S_K417	NPDAD**K**GPWCFTTDPSVR	NVI**K**DNATFSR	plasminogen	streptolysin O	14.6 (2nd)	**b (DSS)**
**7)**, P_K528-S_K540	**K**NYCR	**K**VIDER	plasminogen	streptolysin O	32.9 (2nd)	

Supplementary Table 3 I Summary of inter-protein cross-links between PLG and SLO
The table presents the identified cross-linked peptide pairs between PLG and SLO, specifying the numbering of cross-links. For each XL peptide pair (Peptide 1 and Peptide 2), the reactive lysine residues are highlighted in bold. The table also denotes the source proteins (Protein 1 and Protein 2) for the respective peptides. The distance between the cross-linked lysine primary amines (Nζ-Nζ) is provided for both top 1 model shown in **Figure 5c**, 2nd model in **Figure 5d**, and AF2M model in **Figure 5a** with a reference to the corresponding MS/MS spectra, which can be found in **Supplementary Data Figure 9** if available.

Dataset	PLG, apo state	PLG, SLO-bound state
**HDX reaction details**	blank: 1X PBS prepared in H_2O, $pH_{(read)}$ = 7.5; labelling buffer: 1X PBS prepared in 100% D_2O, $pH_{(read)}$ = 7.1; deuterium percentage: 75%; labelling temperature: 4°C; quenching temperature: 4°C	
**HDX time course**	0 s, 30 s, 300 s, 3000 s, 9000 s	
**HDX controls**	reference protein (Phosphorylase b kinase, 1237 residues) used to check the consistency and accuracy of experimental conditions and instrumentation	
**Back-exchange**	N/A	
**Number of peptides**	150 peptides used for analysis in both states	
**Sequence coverage**	66.12 %	
**Average peptide length/redundancy**	11.70 / 3.36	
**Replicates (biological or technical)**	3 replicates applied per condition per deuterium incorporation time point	
**Repeatability**	0.0560 (average SD) for apo state; repeatability: 0.0516 (average SD) for SLO-bound state	
**Significant differences in HDX**	peptide-level FDR-controlled significance test: p-value < 0.001<br>globally estimated hybrid significance test: p-value < 0.001 + confidence intervals of 0.66 Da	

Supplementary Table 4 | HDX-MS data summary

**Cross-linker**			
**Name**	DSS-H12/D12	DSG-H6/D6	
**Elemental composition**	C16 H20 N2 O8 /<br>C16 D12 H8 N2 O8	C13 H14 N2 O8 /<br>C13 D6 H8 N2 O8	
**Molecular weight (g/mol)**	368 / 380	326 / 332	
**solvent**	DMF	DMF	
**Spacer length (Å)**	11.4	7.7	
**Maximum Cα-Cα distance (Å)**	26–30	22-26	
**Amino acid target (site 1)**	primarily K, protein N-term	primarily K, protein N-term	
**Amino acid target (site 2)**	primarily K, protein N-term	primarily K, protein N-term	
**Amino acid target (site 3)**	none	none	
**Modification introduced by cross-linking by DSS-H12/D12**			
	**Elemental composition**	**Mass difference (Da)**	
**Type 0 (hydrolysed)**	C8 H12 O3 /<br>C8 D12 O3	156.07864 /<br>168.15437	
**Type 0 (quenching)**	C8 H13 O2 N1 /<br>C8 D12 H1 O2 N1	155.09462 /<br>167.17035	
**Type 1 (intra-link)**	C8 H12 O3 /<br>C8 D12 O3	156.07864 /<br>168.15437	
**Type 2 (inter-link)**	C8 H10 O2 /<br>C8 D12 H(-2) O2	138.06807 /<br>150.1438	
**Modification introduced by cross-linking by DSG-H6/D6**			
	**Elemental composition**	**Mass difference (Da)**	
**Type 0 (hydrolysed)**	C5 H6 O3 /<br>C5 D6 O3	114.03169 /<br>120.07537	
**Type 0 (quenching)**	C5 H7 O2 N /<br>C5 D6 H1 O2 N	113.04768 /<br>119.08534	
**Type 1 (intra-link)**	C5 H6 O3 /<br>C5 D6 O3	114.03169 /<br>120.07537	
**Type 2 (inter-link)**	C5 H4 O2 /<br>C5 D6 H(-2) O2	96.02113 /<br>102.06481	
**PLG (plasminogen)**		**SLO (streptolysin O)**	
**Supplier**	Sigma-Aldrich	**Supplier**	Sigma-Aldrich
**UniProt accession no.**	P00747	**UniProt accession no.**	P0DF96
**Buffer**			
**Name**	**Concentration**	**pH**	**Temperature**
Phosphate buffered saline	1 X	7.4	RT
sodium chloride	150 mM		
phosphate	10 mM		
DTT	none		
Glycerol (v/v)	none		
…	none		
**Cross-linking reaction conditions**			
**Cross-linker concentration (μM)**	1000		
**Protein concentration**	0.165 mg/ml		
**Temperature**	37°C		
**Reaction time**	120 min		
**DMF final concentration (v/v)**	2 %		

Quenching			
**Name**	**Concentration (mM)**	**Temperature**	**Incubation time (min)**
Ammonium bicarbonate	50	37°C	30

SDS-PAGE (%) of cross-linked proteins under different conditions

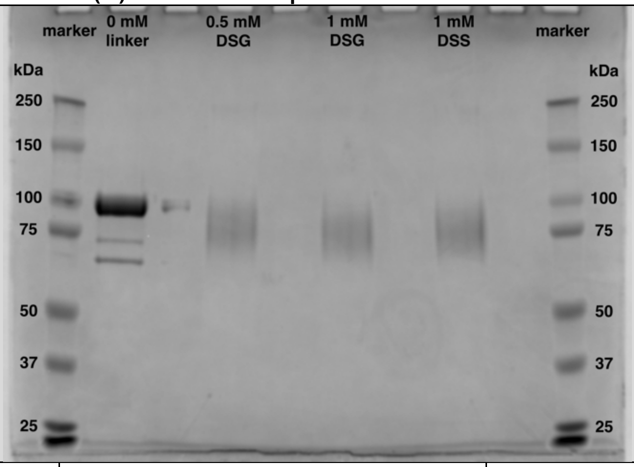

**Sample preparation (in-solution digestion)**			
	**Denaturation and reduction**		
1st step	**Concentration**	**Temperature (°C)**	**Incubation time (h)**
UREA	6 M	37	1
TCEP	1 mM	37	1
	**Alkylation**		
2nd step	**Concentration**	**Temperature (°C)**	**Incubation time (h)**
IAA	10 mM	RT	0.5 (in the dark)
3rd step	**2-step digestion scheme**		
	**Enzyme/protein ratio (w/w)**	**Temperature (°C)**	**Incubation time (h)**
Lysyl Endopeptidase®, Mass Spectrometry Grade (Lys-C)	1:20	37	2
Sequencing Grade Modified Trypsin	1:10	37	overnight (16-18 h)
4th step	**Acidification and peptide clean-up**		
See at Methods section	Add 10% formic acid till pH 3	C18 clean-up spin column	
**LC-MS set-up and acquisition methods**: see at Methods section			
**Data analysis, replicates, Venn diagrams**			
**Raw data conversion**	MSConvert / built-in function	**Replicate overlap *pLink 2 searching**	
**Software**	pLink 2 / MaxLynx; TX-MS MS/MS analysis	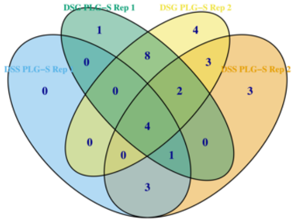	unique interprotein XL sites between PLG and SLO
**Weblink**	http://pfind.org/software/pLink/index.html ; https://maxquant.net/maxquant/ ; http://www.txms.org/index.html		
**a.a. target (site 1)**	K, protein N-term		
**a.a. target (site 2)**	K, protein N-term		
**a.a. target (site 3)**	none		
**Enzyme specificity**	R, K		
**Missed cleavages**	R , K (max 2 miss cleavages in any combination		
**Static modifications**	Carbamidomethylation (C)		
**Variable modifications**	Oxidation (M), Acetyl (protein N-term)		
**Peptide length**	6-60		
**Charge state**	2-6		
**Mass accuracy (precursor ion)**	Filtered precursor error distribution (ppm): $\mu = -0.41$, $\sigma = 1.97$		
**FDR**	pLink 2: Separate FDR ≤ 0.05 at PSM level; MaxLynx: 0.01 FDR at CSM level		

**Decoy analysis**	Yes	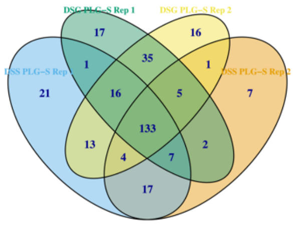	unique intraprotein XL sites within PLG
**Manual validation**	Yes	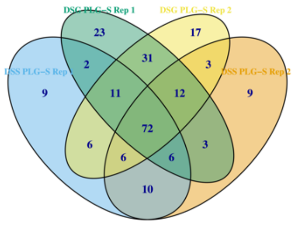	unique intraprotein XL sites within SLO

Supplementary Table 5 l XL-MS data summary

Supplementary Notes

>baitlTagged_GFPlTagged_GFP GFP control bait, Strep-HA-hexaHis tagged at N-terminus
MHHHHHHYPYDVPDYAWSHPQFEKENLYFQSMARKGEELFTGVVPILVELDGDVNGHKFSVRGEGEGDATNGKLTLKFICTTGKLP
VPWPTLVTTLTYGVQCFARYPDHMKQHDFFKSAMPEGYVQERTISFKDDGTYKTRAEVKFEGDTLVNRIELKGIDFKEDGNILGHKL
EYNFNSHNVYITADKQKNGIKANFKIRHNVEDGSVQLADHYQQNTPIGDGPVLLPDNHYLSTQSVLSKDPNEKRDHMVLLEFVTAAGI
THGMDELYK

>baitlTagged_SLOlTagged_SLO Spy Bait protein, Strep-HA-hexaHis tagged at N-terminus.
reference UniProtID: P0DF97 (TACY_STRPQ)
MHHHHHHYPYDVPDYAWSHPQFEKENLYFQSMNKQNTASTETTTTNEQPKPESSELTTEKAGQKTDDMLNSNDMIKLAPKEMPLE
SAEKEEKKSEDKKKSEEDHTEEINDKIYSLNYNELEVLAKNGETIENFVPKEGVKKADKFIVIERKKKNINTTPVDISIIDSVTDRTYPAAL
QLANKGFTENKPDAVVTKRNPQKIHIDLPGMGDKATVEVNDPTYANVSTAIDNLVNQWHDNYSGGNTLPARTQYTESMVYSKSQIE
AALNVNSKILDGTLGIDFKSISKGEKKVMIAAYKQIFYTVSANLPNNPADVFDKSVTFKELQRKGVSNEAPPLFVSNVAYGRTVFVKLE
TSSKSNDVEAAFSAALKGTDVKTNGKYSDILENSSFTAVVLGGDAAEHNKVVTKDFDVIRNVIKDNATFSRKNPAYPISYTSVFLKNN
KIAGVNNRTEYVETTSTEYTSGKINLSHQGAYVAQYEILWDEINYDDKGKEVITKRRWDNNWYSKTSPFSTVIPLGANSRNIRIMARE
CTGLAWEWWRKVIDERDVKLSKEINVNISGSTLSPYGSITYK

>baitlTagged_SCPAlTagged_SCPA Spy Bait protein, Strep-HA-hexaHis tagged at C-terminus.
reference UniProtID: P15926 (C5AP_STRPY)
MNTVTEDTPVTEQAVETPQPTAVSEEVPSSKETKTPQTPDDAEETIADDANDLAPQAPAKTADTPATSKATIRDLNDPSQVKTLQEK
AGKGAGTVVAVIDAGFDKNHEAWRLTDKTKARYQSKEDLEKAKKEHGITYGEWVNDKVAYYHDYSKDGKTAVDQEHGTHVSGILS
GNAPSETKEPYRLEGAMPEAQLLLMRVEIVNGLADYARNYAQAIRDAVNLGAKVINMSFGNAALAYANLPDETKKAFDYAKSKGVSI
VTSAGNDSSFGGKTRLPLADHPDYGVVGTPAAADSTLTVASYSPDKQLTETAMVKTDDQQDKEMPVLSTNRFEPNKAYDYAYANR
GMKEDDFKDVKGKIALIERGDIDFKDKVANAKKAGAVGVLIYDNQDKGFPIELPNVDQMPAAFISRKDGLLLKDNPQKTITFNATPKVL
PTASGTKLSRFSSWGLTADGNIKPDIAAPGQDILSSVANNKYAKLSGTSMSAPLVAGIMGLLQKQYETQYPDMTPSERLDLAKKVLM
SSATALYDEDEKAYFSPRQQGAGAVDAKKASAATMYVTDKDNTSSKVHLNNVSDKFEVTVTVHNKSDKPQELYYQATVQTDKVDG
KHFALAPKVLYEASWQKITIPANSSKQVTVPIDASRFSKDLLAQMKNGYFLEGFVRFKQDPTKEELMSIPYIGFRGDFGNLSAVEKPIY
DSKDGSSYYHEANSDAKDQLDGDGLQFYALKNNFTALTTESNPWTIIKAVKEGVENIEDIESSEITETIFAGTFAKQDDDSHYYIHRHA
NGEPYAAISPNGDGNRDYVQFQGTFLRNAKNLVAEVLDKEGNVVWTSEVTEQVVKNYNNDLASTLGSTRFEKTRWDGKDKDGKVV
ANGTYTYRVRYTPISSGAKEQHTDFDVIVDNTTPEVATSATFSTEDRRLTLASKPKTSQPVYRERIAYTYMDEDLPTTEYISPNEDGTF
TLPEEAETMEGATVPLKMSDFTYVVEDMAGNITYTPVTKLLEGHSENLYFQSWSHPQFEKYPYDVPDYAHHHHHH
