# Supplementary material for: Streptolysin O accelerates the conversion of plasminogen to plasmin
Source: Nat Commun. 2024 Nov 25;15:10212. doi: 10.1038/s41467-024-54173-6 (PMC11589678; doi:10.1038/s41467-024-54173-6)
Supplement: Supplementary file 2 — Reporting Summary [file 41467_2024_54173_MOESM2_ESM.pdf]

nature portfolio

Corresponding author(s): Di Tang, Johan Malmström

Last updated by author(s): Oct 14, 2024

Reporting Summary

Nature Portfolio wishes to improve the reproducibility of the work that we publish. This form provides structure for consistency and transparency in reporting. For further information on Nature Portfolio policies, see our Editorial Policies and the Editorial Policy Checklist.

Statistics

For all statistical analyses, confirm that the following items are present in the figure legend, table legend, main text, or Methods section.

n/a	Confirmed	
☐	☒	The exact sample size (*n*) for each experimental group/condition, given as a discrete number and unit of measurement
☐	☒	A statement on whether measurements were taken from distinct samples or whether the same sample was measured repeatedly
☐	☒	The statistical test(s) used AND whether they are one- or two-sided<br>*Only common tests should be described solely by name; describe more complex techniques in the Methods section.*
☒	☐	A description of all covariates tested
☐	☒	A description of any assumptions or corrections, such as tests of normality and adjustment for multiple comparisons
☐	☒	A full description of the statistical parameters including central tendency (e.g. means) or other basic estimates (e.g. regression coefficient) AND variation (e.g. standard deviation) or associated estimates of uncertainty (e.g. confidence intervals)
☐	☒	For null hypothesis testing, the test statistic (e.g. *F*, *t*, *r*) with confidence intervals, effect sizes, degrees of freedom and *P* value noted<br>*Give P values as exact values whenever suitable.*
☒	☐	For Bayesian analysis, information on the choice of priors and Markov chain Monte Carlo settings
☒	☐	For hierarchical and complex designs, identification of the appropriate level for tests and full reporting of outcomes
☒	☐	Estimates of effect sizes (e.g. Cohen's *d*, Pearson's *r*), indicating how they were calculated

Our web collection on statistics for biologists contains articles on many of the points above.

Software and code

Policy information about availability of computer code

Data collection	LC-MS and LC-MS/MS data acquisition software packages provided by Thermo Fisher Scientific Inc.. GAS genomes sourced from The Bacterial and Viral Bioinformatics Resource Center (https://www.bv-brc.org/)
Data analysis	Maxquant (v.2.0.3.0); Perseus (v.1.6); MiST scoring pipeline (v.1.5); pGlyco (v.3.0); Cytoscape (v.3.9); STRING (v.2.0.0, accessed in Apr.2022) GraphPad Prism software (v.9.0;v.10.0); Metascape (v.3.5); R version (v.4.2); ggplot2 (v.3.5.1); pheatmap (v.1.0.12); ggfortify (v.0.4.17); circlize (v.0.4.16); PEAKS Studio (v.8.5); HDExaminer (v.3.3.0); Deuteros (v.2.4.2); pLink 2 (v.2.3.10); MaxLynx (v.2.0.4); xiVIEW (xiview.org); XL Mapping and AnalySis (XMAS) with UCSF ChimeraX (v.1.6.1;v.1.8); XlinkCyNET (v.1.5.2).

For manuscripts utilizing custom algorithms or software that are central to the research but not yet described in published literature, software must be made available to editors and reviewers. We strongly encourage code deposition in a community repository (e.g. GitHub). See the Nature Portfolio guidelines for submitting code & software for further information.

Data

Policy information about availability of data

All manuscripts must include a data availability statement. This statement should provide the following information, where applicable:

- Accession codes, unique identifiers, or web links for publicly available datasets
- A description of any restrictions on data availability
- For clinical datasets or third party data, please ensure that the statement adheres to our policy

All mass spectrometry proteomics data have been deposited to the ProteomeXchange Consortium via the PRIDE partner repository with the dataset identifier

PXD051261. Source data are provided with this manuscript.

Research involving human participants, their data, or biological material

Policy information about studies with human participants or human data. See also policy information about sex, gender (identity/presentation), and sexual orientation and race, ethnicity and racism.

Reporting on sex and gender	n/a
Reporting on race, ethnicity, or other socially relevant groupings	n/a
Population characteristics	n/a
Recruitment	n/a
Ethics oversight	n/a

Note that full information on the approval of the study protocol must also be provided in the manuscript.

Field-specific reporting

Please select the one below that is the best fit for your research. If you are not sure, read the appropriate sections before making your selection.

[x] Life sciences [ ] Behavioural & social sciences [ ] Ecological, evolutionary & environmental sciences

For a reference copy of the document with all sections, see nature.com/documents/nr-reporting-summary-flat.pdf

Life sciences study design

All studies must disclose on these points even when the disclosure is negative.

Sample size	Sample sizes were determined according to either significance level α (0.01), the desired power 1 – β (0.8/0.7) and an effect size greater than a 2 fold change, or community-standard practice in structural proteomics, biochemical assays and computational modelling. Relevant citation protocol: Schork, K., Podwojski, K., Turewicz, M., Stephan, C., Eisenacher, M. (2021). Important Issues in Planning a Proteomics Experiment: Statistical Considerations of Quantitative Proteomic Data. In: Marcus, K., Eisenacher, M., Sitek, B. (eds) Quantitative Methods in Proteomics. Methods in Molecular Biology, vol 2228. Humana, New York, NY. https://doi.org/10.1007/978-1-0716-1024-4_1
Data exclusions	No data were excluded from the analysis.
Replication	All attempts at replication were successful. Information regarding experimental replicates and technical replicates have been provided in corresponding Figure legends and Methods section.
Randomization	The purpose of our study is to identify and characterise new binders to a specific bait protein from human plasma mixture, focusing on discovery rather than testing causal hypotheses. Therefore, randomization is not relevant. For the detailed validation of the bait-prey protein interaction, randomization is also unnecessary. The mechanistic nature of our validation, controlled conditions, rigorous replication, and advanced analytical techniques provide a robust and reliable framework, consistent with established scientific protocols.
Blinding	Blinding is not applicable because our study relies on objective, measurable data obtained through a combination of analytical techniques and controlled conditions, ensuring reliable results without bias.

Reporting for specific materials, systems and methods

We require information from authors about some types of materials, experimental systems and methods used in many studies. Here, indicate whether each material, system or method listed is relevant to your study. If you are not sure if a list item applies to your research, read the appropriate section before selecting a response.

Materials & experimental systems

n/a	Involved in the study	
[ ]	[x]	Antibodies
[x]	[ ]	Eukaryotic cell lines
[x]	[ ]	Palaeontology and archaeology
[x]	[ ]	Animals and other organisms
[x]	[ ]	Clinical data
[x]	[ ]	Dual use research of concern
[x]	[ ]	Plants

Methods

n/a	Involved in the study	
[x]	[ ]	ChIP-seq
[x]	[ ]	Flow cytometry
[x]	[ ]	MRI-based neuroimaging

Antibodies

Antibodies used	anti-streptolysin O antibody, sourced from Abcam, catalog number ab23501, clone number 6D11, lot number GR3430639-3; 105629-1; P.IgG, a polyclonal IgG mixture isolated from a donor, has its source detailed in the ethics statement.
Validation	https://www.abcam.com/en-se/products/primary-antibodies/streptolysin-antibody-6d11-ab23501#

Plants

Seed stocks	n/a
Novel plant genotypes	n/a
Authentication	n/a
